# The Black Box of Cellular and Molecular Events of *Plasmodium vivax* Merozoite Invasion into Reticulocytes

**DOI:** 10.3390/ijms232314528

**Published:** 2022-11-22

**Authors:** Jessica Molina-Franky, César Reyes, Yelson Alejandro Picón Jaimes, Markus Kalkum, Manuel Alfonso Patarroyo

**Affiliations:** 1 Department of Immunology and Theranostics, Arthur Riggs Diabetes and Metabolism Research Institute, Beckman Research Institute of the City of Hope, Duarte, CA 91010, USA; 2 Molecular Biology and Immunology Department, Fundación Instituto de Inmunología de Colombia (FIDIC), Bogotá 112111, Colombia; 3 Biotechnology, Faculty of Sciences, Universidad Nacional de Colombia, Bogotá 111321, Colombia; 4 Animal Sciences Faculty, Universidad de Ciencias Aplicadas y Ambientales (U.D.C.A), Bogotá 111166, Colombia; 5 Faculty of Health Sciences, Universidad del Alba, Santiago 8320000, Chile; 6 Faculty of Medicine, Universidad Nacional de Colombia, Bogotá 111321, Colombia

**Keywords:** malaria, *Plasmodium vivax*, merozoite, reticulocyte, host cell invasion

## Abstract

*Plasmodium vivax* is the most widely distributed malaria parasite affecting humans worldwide, causing ~5 million cases yearly. Despite the disease’s extensive burden, there are gaps in the knowledge of the pathophysiological mechanisms by which *P. vivax* invades reticulocytes. In contrast, this crucial step is better understood for *P. falciparum,* the less widely distributed but more often fatal malaria parasite. This discrepancy is due to the difficulty of studying *P. vivax*’s exclusive invasion of reticulocytes, which represent 1–2% of circulating cells. Its accurate targeting mechanism has not yet been clarified, hindering the establishment of long-term continuous in vitro culture systems. So far, only three reticulocyte invasion pathways have been characterised based on parasite interactions with DARC, TfR1 and CD98 host proteins. However, exposing the parasite’s alternative invasion mechanisms is currently being considered, opening up a large field for exploring the entry receptors used by *P. vivax* for invading host cells. New methods must be developed to ensure better understanding of the parasite to control malarial transmission and to eradicate the disease. Here, we review the current state of knowledge on cellular and molecular mechanisms of *P. vivax’*s merozoite invasion to contribute to a better understanding of the parasite’s biology, pathogenesis and epidemiology.

## 1. Introduction

Malaria is a major public health problem, causing an estimated 241 million cases and over 627,000 deaths in 2020 [1]. Six *Plasmodium* species cause malaria in humans: *P. falciparum*, *P. vivax*, *P. malariae*, *P. knowlesi*, *P. ovale wallikeri* and *P. ovale curtisi*. *P. falciparum* is the deadliest species and *P. vivax* the most widely distributed, being most prevalent in South America, India and Southeast Asia. *P. vivax* was found to be largely incapable of infecting part of the west African population as a result of a silent mutation in the Duffy blood group (Fy(a−b−)) disabling the Duffy antigen receptor for chemokines (DARC) interaction with the *P. vivax* Duffy binding protein (*Pv*DBP), thereby conferring resistance against such infection [2]. However, cases involving this malarial species have been increasingly reported in Duffy-negative populations during the last few years (Table 1).

**Figure 1:**
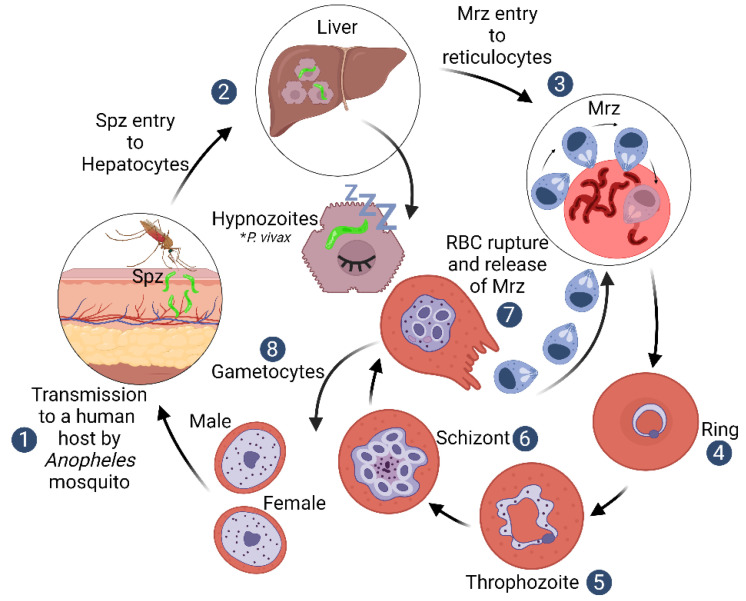
*Plasmodium vivax* life cycle. The life cycle of human malaria begins when a female *Anopheles* mosquito inoculates sporozoites (Spz) into a host’s dermis through its proboscis; these Spz invade hepatocytes in the liver and some of them remain as dormant liver-stage parasites–hypnozoites [10]. Each Spz forms thousands of Mrz that are released into the bloodstream and invade RBCs through specific receptor–ligand protein interactions, initiating a sequence of molecular events that commit the parasite to cell invasion [4]. The parasites undergo mitotic division and cytoplasmic growth during the next ~48 h inside the RBC to develop either directly into schizont (asexual) or gametocyte forms (sexual) [11].

An in vitro *P. falciparum* culture was developed more than 40 years ago [3]. Several cellular and molecular interactions between invading merozoites (Mrz) and the targeted erythrocytes have been elucidated since its establishment. Information regarding multi-step *P. vivax* Mrz entry into reticulocytes has been largely taken from such studies; however, there is still a considerable gap in relevant knowledge to be explored. *P. vivax* Mrz invasion of red blood cells (RBCs) is a rapid, coordinated process (less than 1 min), orchestrated by specific receptor–ligand interactions. It begins with initial Mrz contact with the RBC membrane and is followed by Mrz apical pole reorientation on reticulocytes. Ligands are released from specialised apical organelles (rhoptries and micronemes) to specifically interact with reticulocyte receptors to enable strong attachment and a subsequent tight junction (TJ) formation. Active parasite invasion occurs through the action of the mobile junction complex and the parasitophorous vacuole (PV) formation [4] (Figure 1).

*P. falciparum*, *P. knowlesi* and *P. malariae* Mrz invade mature erythrocytes, although *P. falciparum* and *P. knowlesi* can also invade reticulocytes [5,6], whilst *P. vivax* and *P. ovale* are restricted to invading reticulocytes [7,8]. Such immature cells represent only 1–2% of circulating RBCs, indicating that these two *Plasmodium* species’ Mrz have an as-yet-unknown mechanism to accurately target the reticulocyte population during invasion.

The biological particularities of *P. vivax* have become a challenge for the development of a long-term continuous in vitro culture system. Different culture conditions, along with reticulocyte and parasite sources, have been tried without success so far, thereby delaying understanding of the parasite and, therefore, the development of mechanisms for controlling or eradicating the disease [9]. This review describes the cellular and molecular mechanisms related to *P. vivax* Mrz invasion of reticulocytes, contributing to a better understanding of the parasite’s biology and pathogenesis.

## 2. *Plasmodium vivax* Biological Particularities

*P. falciparum*-related clinical complications have also been reported for *P. vivax*, including haemolytic anaemia, coagulation disorders, acute respiratory distress syndrome, nephropathology, porphyria, rhabdomyolysis and cerebral malaria [12]. However, *P. vivax* has a different evolutionary path to that of *P. falciparum*, being more closely related to *P. cynomolgi* (a species infecting Asian macaque monkeys) [13].

*P. vivax* has a hypnozoite stage where parasites can remain dormant for long periods in host hepatocytes (Figure 1). This produces relapses in a host when they are released into the bloodstream weeks or months later (the mechanism of these kinds of reactivations remains unknown) [10]. The *P. vivax* life cycle blood stage involves a restriction to exclusively invading reticulocytes. The parasite’s mechanisms enabling such an exclusive invasion tropism have not yet been elucidated [7]. Gametocytes are produced early on during infection after Mrz have been released from infected RBCs, which is very different from the life cycle of *P. falciparum*, where gametocytes are not found in peripheral blood until after multiple blood-stage cycles. The latter is due to a requirement for bone marrow sequestration for *P. falciparum*’s gametogenesis, which takes 10 to 12 days to form fully transmissible gametocytes [14]. These distinctive *P. vivax* life cycle characteristics represent a major challenge for understanding *P. vivax* pathogenesis and for controlling the disease worldwide.

## 3. *Plasmodium vivax* Mrz Exclusive Invasion of Reticulocytes

Reticulocytes are immature RBCs produced in host bone marrow which become released into peripheral blood, where they become normocytes within one to two days during erythropoiesis; this produces two million RBCs every second in a healthy human adult [15]. These cells undergo multiple structural changes until they mature. The erythropoietic process starts within the bone marrow, where haematopoietic stem cells are subjected to chromatin and nuclear condensation. They then interact with macrophages, forming reticulocytes, thereby enabling enucleation to take place [16].

Organelles, such as endoplasmic reticulum, Golgi apparatus, lysosomes, mitochondria and ribosomes, are removed via autophagic and non-autophagic pathways, beginning in the bone marrow and continuing in the bloodstream. RNA breakdown is facilitated by ribonucleases; modifications in cell volume and membrane remodelling are thought to occur via exosomes during haematopoiesis. Cells become smaller, more condense and produce specific proteins such as haemoglobin, until mature biconcave RBCs are created at the culmination of terminal maturation [15].

Heilmeyer first classified reticulocyte cells according to their microscopic appearance after New Methylene Blue staining [17]. They were then classified as R1 or R2 based on their shape and movement in live-cell cytology studies. R1 reticulocytes have expelled their nuclei in bone marrow, retain their residual reticulum and are motile and multi-lobular, whilst R2 in peripheral blood are non-motile and mechanically stable [18]. The latter proposed classification depends on the amount of transferrin receptor 1 (TfR1, also known as cluster of differentiation 71 or CD71) expression on the cell membrane [19], based on the progressive loss of this membrane protein during maturation by exocytosis, until they become completely absent in mature RBCs [19]. Accordingly, CD71 has proved to be a useful biomarker for the fine-scale age grading of reticulocytes.

A healthy adult has Heilmeyer stage IV reticulocytes (CD71^low^) and normocytes (CD71^-^) circulating in bone marrow; Heilmeyer stage I-III reticulocytes have higher CD71 concentrations. *P. vivax* has been shown to have a predilection for invading reticulocyte stages I-III [7].

Reticulocyte maturation involves significant heterogeneity in terms of morphology, cell content and protein expression, and represents a population in constant phenotypical change until they become normocytes, which makes collecting enriched samples for in vitro cultures more challenging. Different methods have been proposed for obtaining enriched reticulocyte samples, i.e., producing erythroid cells lines [20,21], umbilical cord blood samples containing 6.9–7.9% reticulocytes [22], blood from non-human primate models [23], humanised mouse models such as human liver chimeric FRG KO huHep [24] and humans with blood disorders, including haemochromatosis or β-thalassaemia [25]. However, the barrier hindering the development of a long-term *P. vivax* continuous in vitro culture system still requires much work to be overcome.

## 4. The Reticulocyte Gateway for *Plasmodium vivax* Mrz

Host–parasite interactions between *P. vivax* ligands and reticulocyte receptors are poorly understood. *P. vivax* tropism for invading reticulocytes expressing DARC and TfR-1 surface receptors has been established [26,27]; however, there is still a long way to go to identify and characterise the receptors expressed on the reticulocyte membrane that serve as an entry point via protein–protein interactions with parasite invasion ligands.

DARC is involved in Mrz invasion and bound by *Pv*DBP1, a 140 kDa protein member of the erythrocyte binding-like (EBL) protein superfamily [26]. Proteins from this family have one or two extracellular cysteine-rich Duffy binding-like (DBL) domains (RII in *Pv*DBP1), an extracellular cysteine-rich domain (region VI in *Pv*DBP1), a transmembrane domain and a short cytoplasmatic domain [26].

*Pv*DBP is characterised by a single 302 residue-long, cysteine-rich DBL located in the protein’s extracellular N-terminus (RII-DBP1); this region has three subdomains (SD1–SD3) stabilised by intra-subdomain disulphide bridges. SD1 (^211^N–L^253^) has two intra-subdomain disulphide bridges, (^217^C–C^246^) and (^230^C–C^237^), whilst SD2 (^271^Y–E^386^) has one intra-subdomain disulphide bridge, (^300^C–C^377^), and SD3 (^387^P–S^508^) has three disulphide bridges, (^415^C–C^432^), (^427^C–C^507^) and (^436^C–C^505^) [26,28].

**Figure 2:**
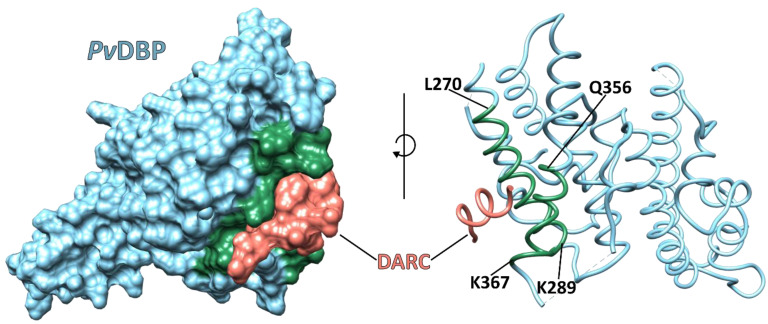
Crystal structure of *Pv*DBP-RII bound to the DARC core region (PDB: 4NUU). The *Pv*DBP-RII region that interacts with the DARC consists of residues 256-426 located in SD2. Binding develops in a stepwise manner to create a stable, two-molecule heterotetramer consisting of two DBP (blue) copies and two molecules of the DARC (salmon). Green represents the *Pv*DBP-RII helices forming the receptor binding pocket [28].

*Pv*DBP1 is contained within the parasite’s apical micronemes during the *P. vivax* Mrz irreversible reticulocyte binding process, followed by its release to interact with its reticulocyte receptor, DARC, via RII (residues 198 to 522). The *Pv*DBP-RII residues that directly contact the receptor are ^270^L–K^289^, ^356^Q–K^367^ and ^261^F–T^266^ (Figure 2) [26,28]. The DARC residues 19–30 are critical for *Pv*DBP-RII binding [28]. The main receptor recognition site lies between cysteines 4 and 7 located in RII-DBP [29], a polymorphic region with a substitution rate four times higher than the rest of the protein (consistent with high immune selection pressure), complicating the development of a malaria vaccine [28,30].

The DARC serves as a receptor for several chemokines, such as melanoma growth-stimulating activity (MGSA) and interleukin (IL)-8; these can inhibit *P. vivax* and *P. knowlesi* binding to DBL domains and Duffy-positive (DARC-positive) erythrocytes, suggesting that the binding sites on the DARC used by chemokines and parasite proteins overlap [2,26]. The DARC is present on both mature normocytes and reticulocytes; interaction with *Pv*DBP therefore does not fully explain the specific *P. vivax* Mrz recognition of reticulocytes. Although it has been proposed that exposure of the *Pv*DBP binding site in the DARC on reticulocytes could enable preferential binding [31], details on such a mechanism have yet to be studied in depth.

*Pv*DBP is a leading vaccine candidate [32]. *Pv*DBP-RII antibodies (Abs) inhibit Mrz invasion of human RBCs by ~80% [33]. Individuals living in *P. vivax*-endemic regions develop natural, age-dependent immunity, which is strongly related to humoral and cellular recognition of *Pv*DBP-RII during natural infection [34,35]. Naturally acquired human Abs 053054 and 092096 target the DARC binding site and dimer interface on *Pv*DBP-RII and inhibit such interaction. The Ab 053054 epitope includes residues E^249^, ^264^D-A^281^ and ^356^Q-N^372^ belonging to subdomain 2 located in RII. This epitope overlaps with the Ab 092096 epitope consisting of residues Y^219^, E^249^, ^270^L-K^289^ and ^356^Q-W^375^ [36]. Monoclonal antibodies (mAb) 3C9, 2D10, 2H2 and 2C6 block *Pv*DBP-RII binding to human erythrocytes, targeting a region located in SD3 [37]. The mAb 3C9 epitope lies between residues 476–493; mAb 2D10 binds to a conformational epitope located between residues 413–417 and 425–441; mAb 2H2 shares overlapping binding regions with 2D10 and 2C6, recognising an epitope between residues 465–485. These Abs do not target the *Pv*DBP–DARC interaction interface directly; it has therefore been suggested that their mechanism of action is based on steric hindrance preventing *Pv*DBP-RII’s approach to the reticulocyte surface. Although the binding interface has been characterised in detail, it has been difficult to create a vaccine targeting such a particular region due to the high polymorphism levels displayed by *Pv*DBP-RII.

A major component of the pathway for *P. vivax* Mrz entry into reticulocytes is mediated by the specific interaction between the reticulocyte-binding protein 2b (*Pv*RBP2b), located in the micronemes, and the host membrane protein TfR1 in a sialic acid-independent manner [27].

TfR1 (or CD71), is a homodimer type II transmembrane glycoprotein of about 170 kDa; it is expressed in large quantities on immature RBCs, is present in up to one million copies/cells in reticulocytes and is gradually lost during RBC maturation until it is completely absent from normocytes [38]. It is thus presumed that it could be an essential factor for accurate *P. vivax* recognition of reticulocytes. TfR1 is present in Duffy-positive and Duffy-negative reticulocytes and it has been supposed that *Pv*RBP2b interaction with TfR1 occurs prior to the *Pv*DBP interaction with the DARC [39]. However, the role of this interaction in Duffy-negative reticulocytes has yet to be studied.

**Figure 3:**
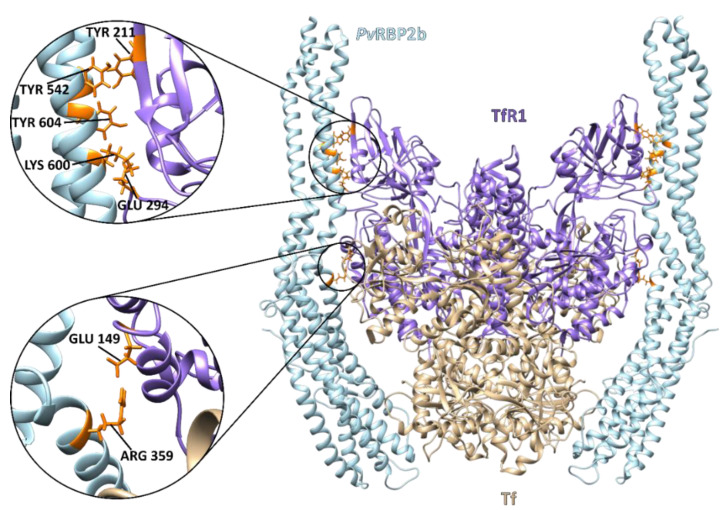
Cryo-electron microscopy structure of the ternary *Pv*RBP2b complex bound to human TfR1 and Tf (PDB: 6D04). The complex consists of homodimer TfR1 (residues 120–760) (purple) bound to two molecules of iron-loaded Tf (residues 1–679) (beige) and two molecules of *Pv*RBP2b (residues 168–633) bound on either side (light blue). The critical *Pv*RBP2b and TfR1 binding residues are enlarged and highlighted in orange [40].

A 3.7 Å resolution cryo-electron microscopy (cryo-EM) structure has reported the critical residues involved in ternary complex interactions between *Pv*RBP2b, TfR1 and Tf (Figure 3). TfR1 binds to its human ligand, iron-loaded transferrin (Tf), and this TfR1/Tf complex regulates one of the main mechanisms for transporting iron into cells [40].

TfR1–*Pv*RBP2b is an essential interaction for the *P. vivax* Mrz invasion of reticulocytes. *Pv*RBP2b binding-deficient TfR1 mutant cells were refractory to *P. vivax* invasion by ~90%, but not to *P. falciparum* invasion [27]. Experiments using Thai and Brazilian *P. vivax* isolates have shown that mouse mAbs 3E9, 6H1 and 10B12 that bound to epitopes within the N-terminal domain in *Pv*RBP2b 169–470 induced a 45–68% inhibition of *P. vivax* invasion. These Abs did not completely overlap with the receptor recognition site but would have either sterically clashed with *Pv*RBP2b (3E9) TfR1-Tf binding or inhibited receptor engagement through steric hindrance with the host cell membrane (in the case of 6H1 and 10B12) [27]. Twenty-two human anti-*Pv*RBP2b mAbs isolated from two individuals in Cambodia suffering natural *P. vivax* infection inhibited *Pv*RBP2b binding to reticulocytes by ~40%-98% and blocked the formation of the *Pv*RBP2b-TfR1–Tf complex [41]. The essentiality of this interaction makes *Pv*RBP2b an attractive vaccine candidate antigen.

Recently, the interaction between the reticulocyte-binding protein 2a (*Pv*RBP2a) with CD98 (K_D_ of 5.9 nM) was described; CD98 is a transmembrane glycoprotein that decreases its expression during RBC maturation from CD71^+^ to CD71^−^ cells [42]. It is thus presumed that, like TfR1, it may be an essential factor for the precise recognition of reticulocytes by *P. vivax* Mrz [42]. CD98 binds to a *Pv*RBP2a region located between residues 23–767, this binding being significantly inhibited by Anti-*Pv*RBP2a 1C3 and 3A11 Abs [42]. Experiments using Thai *P. vivax* isolates have shown that Anti-CD98 Abs inhibited *P. vivax* Mrz invasion of CD71+ reticulocytes by ~70% [42]. The *Pv*RBP2a–CD98 route is now considered a major ligand receptor involved in the *P. vivax* invasion of reticulocytes, drawing attention to this parasite’s protein for further study as a *P. vivax* vaccine candidate.

## 5. *Plasmodium vivax* Infections in the DARC-Negative Population

The DARC is a seven-transmembrane, glycosylated protein consisting of an extracellular N-terminal domain, seven transmembrane domains and a short cytosolic C-terminal domain. It is expressed in reticulocytes and mature erythrocytes (five thousand copies/cells); its expression does not change during RBC maturation [2,43].

The gene encoding the Duffy blood group protein (*Fy*, *DARC* or *ACKR1*) is localised on chromosome 1, specifically at 1q23.2. The Duffy glycoprotein has two major alleles, FY*A (encoding antigen Fya) and FY*B (Fyb) [44]. Genotypes FY*A/FY*A, FY*B/FY*B and FY*A/FY*B give the Duffy-positive phenotypes Fy(a+b-), Fy(a-b+) and Fy(a+b+), respectively [45], whilst the Duffy-negative phenotype Fy(a-b-) is produced by a single-point mutation in the gene promoter GATA-1 transcription factor binding site at position 67T > C [46]. This genotype is known as erythrocyte-silent (ES). Individuals homozygous for this mutation lack the Duffy protein on erythrocytes, whilst those heterozygous for it have ~50% levels [47]. A molecular epidemiology study using Ethiopian *P. vivax* isolates determined that the percentage of parasites with a positive qPCR amplification of *Pv*DBP was higher in individuals with the FY*A allele than in FY*B individuals [48].

The Duffy-negative phenotype is rare amongst Caucasian and Asian populations, whereas it is predominant in sub-Saharan African populations, occurring in over two-thirds of the population [49]. For many years, it was assumed that *P. vivax* infections did not exist in African populations negative for the Duffy blood group antigen [50]. However, many cases of *P. vivax* infection have been reported in Duffy-negative populations during the last decade, even though the level of infection was low and potentially difficult to diagnose without advanced techniques such as a *P. vivax-*specific polymerase chain reaction (PCR). The first report of *P. vivax* infection in the Duffy-negative population came out of Kenya in 2006; it is worth noting that these infections were detected once molecular tools, such as nested PCR and sequencing, had become available for identifying extremely low levels of *P. vivax* infection [51]. Table 1 summarises reports of *P. vivax* in the Duffy-negative population, highlighting the diagnostic methods used.

The question how *P. vivax* Mrz invade Duffy-negative reticulocytes remains. It could be explained by considering alternative invasion pathways, as observed for *P. falciparum*. Whole-genome sequencing (WGS) identified a duplication of the *Pv*DBP gene in samples of Duffy-negative *P. vivax-*infected patients from Madagascar [52]. It was speculated that such gene duplication evolved in response to the Duffy-negative antigen barrier, perhaps by increasing the amount of *Pv*DBP ligand on the Mrz surface for binding to a yet-unknown, low-affinity receptor on the reticulocyte membrane. Against this first hypothesis, a study using WGS of *P. vivax* isolates from around the world indicated that ~30% of Cambodian parasites (Duffy-positive individuals) had *Pv*DBP duplications [48]. Furthermore, quantitative PCR did not reveal differences in the *Pv*DBP copy numbers for Cambodian and Malagasy parasites, suggesting that the Duffy-negative phenotype did not lead to an increased *Pv*DBP copy number [53]. A significant variation in invasion was observed by blocking *Pv*DBP–DARC and *Pv*RBP2b–TfR1 interactions in short-term *P. vivax* isolate cultures with the MGSA cytokine and monoclonal anti-TfR1 antibody (OKT-9), respectively [54]; however, *Pv*DBP itself was not able to bind to Duffy-negative RBC [26,55]. A second hypothesis is based on the finding that FY expression has been described on FY^ES^ RBC erythroid precursor cells, suggesting that the GATA-1 SNP did not completely eliminate Fy expression [56], leading to a leaky DARC expression on RBCs [57]. Nevertheless, there has been no clarification as of now, and these two hypotheses need further investigation.

**Table 1:** X. *P. vivax* infection in Duffy-negative population.

Population	Diagnostic Tools		Results	Year	Ref.
	*P. vivax* Infection	Duffy Genotyping			
17,972 adults from the Democratic Republic of the Congo	PCR, nested-PCR	qPCR and sequencing	*P. vivax* was detected in 467 samples, 464 of which were Duffy-negative.	2021	[58]
107 Duffy-negative and 305 Duffy-positive individuals in Ethiopia and Sudan	PCR	-	412 *P. vivax* positive samples. 16/107 of these were Duffy-negative and 42/305 were Duffy-positive	2021	[59]
Febrile patients from Dshang (n:500), Santchou (n:400) and Kyé-ossi (n:100) in Cameroon	Nested PCR, sequencing	PCR-RFLP	*P. vivax* malaria was detected in 177 Duffy-negative population samples from Dshang, two from Santchou and two from Kyé-ossi.	2021	[60]
1215 febrile patients from Botswana, Ethiopia and the Sudan	PCR	qPCR and sequencing	21 patients in Botswana were positive for *P. vivax*; 18 of these were Duffy-negative. 210 patients in Ethiopia were *P. vivax*-positive, 24 of them being Duffy-negative. 101 patients in the Sudan were *P. vivax-*positive and seven Duffy-negative.	2021	[61]
33 participants infected with *Plasmodium* spp. from Namibia	PCR and sequencing	PCR and sequencing	Three cases of *P. vivax* monoinfection were identified inDuffy-negative individuals from Namibia	2021	[62]
242 individuals form Nigeria	Microscopy, RDT, PCR and sequencing	Sequencing	*P. vivax* infection was identified in four Nigerian isolates, either as single (three) or mixed (one) with *P. falciparum*. All *P. vivax* isolates were Duffy-negative.	2020	[63]
42 *P. vivax-*infected blood samples collected from patients from different areas of Sudan	Rapid diagnostic kit (RDT), microscopy, nested PCR	Nested PCR, sequencing	7 (16.7%) of the 42 Sudanese patients tested for *P. vivax* were Duffy-negative.	2018	[64]
48 school children in the Kedougou region of south-eastern Senegal	Nested PCR	Sequencing	All Senegalese schoolchildren were classified as Duffy-negative. *P. vivax* infection was detected in 20.3% (15/74) of them.	2018	[65]
292 samples from children in the Democratic Republic of the Congo	Nested PCR, species-specific PCR, sequencing	Nested PCR, sequencing	14 *P. vivax* infections were identified in Congolese children; nine were coinfected with *P. falciparum*. All 14 individuals were confirmed as Duffy-negative.	2018	[66]
436 febrile patients from Nigeria	Microscopy, RDT and PCR	Sequencing	Five *P. vivax* infections were identified (four mixed with another *Plasmodium spp.*) in Nigerian patients. All *P. vivax* isolates were Duffy-negative.	2018	[67]
300 blood samples from 0- to 6-year-old children in Bandiagara, a Sahelian area of Mali, West Africa	Microscopy, qPCR, real-time PCR	PCR and sequencing	25 cases of *P. vivax* malaria were identified in Malian patients. All *P. vivax* infections were found in Duffy-negative children.	2017	[68]
992 microscope-positive malaria samples collected from central, northern and eastern parts of Sudan	Microscopy and nested PCR	PCR and sequencing	186 *P. vivax* monoinfections and four mixed *P. vivax* and *P. falciparum* infections were identified in Sudan; 129 (67.9%) of them were Fy(a-b+), 14.2% Fy(a+b-) and 17.9% Fy(a-b-).	2017	[69]
484 individuals from Cameroon	PCR and sequencing	PCR-melting curve analysis	*P. vivax* infection was detected in 5.6% of Cameroon individuals (n = 27/484), all having the Duffy-negative genotype.	2017	[70]
126 patients suspected of having malaria in the Wad Madani hospital in Gezira State, central Sudan	Microscopy, RDT and PCR	PCR-RFLP	48 (38%) Sudanese patients were *P. vivax*-positive and four (8.3%) Duffy-negative.	2016	[71]
1234 healthy blood donors in Benin	Microscopy, RDT and nested PCR	Sequencing	84 samples from Benin were selected for nested PCR analysis, 13 being *P. vivax*-positive and all Duffy-negative.	2016	[72]
60 malaria symptomatic patients from Cameroon	Nested PCR	Sequencing	43/60 were found to be infected with malaria, 33 (76.7%) were due to *P. falciparum* and 10/60 (17%) were *P. vivax* monoinfections. All 10 were Duffy-negative.	2016	[73]
485 malaria-symptomatic patients attending hospitals located in five areas of southern Cameroon	PCR	PCR and sequencing	Only 201 of the 485 samples from Cameroon were infected by malarial parasites. 93 (96%) were due to *P. falciparum*, six (3%) to *P. vivax* and two cases (1%) to mixed parasites. The eight native Cameroonians infected with *P. vivax* were Duffy-negative.	2014	[74]
269 samples from Bolifamba (a rural, multi-ethnic environment, 530 m above sea level), located on the eastern slope of Mount Cameroon	Microscopy, nested PCR, rapid card assay and sequencing	PCR and sequencing	Overall parasite prevalence in the 269 Cameroonians was 32.3%. 14.9% (13/87) of the infections were caused exclusively or concomitantly by *P. vivax*. 50% of thosemonoinfected by *P. vivax* (6/12) were Duffy-negative.	2014	[75]
160 *P. vivax* malaria patients and 160 control individuals from the south-east of Iran	Microscopy	PCR-RFLP, sequencing	160 *P. vivax-*positive samples. 2/6 of these were Duffy-negative and 158/314 were Duffy-positive	2014	[76]
1304 febrile patients from Harar (Jenela) health centre in eastern Ethiopia and 627 from Jimma health centre in southwestern Ethiopia	Microscopy and PCR	Sequencing	74/98 (76%) *P. vivax* cases were identified in Harar, Ethiopia, and 70/107 (65%) *P. falciparum* cases in Jimma. 17/98 (17%) of these were reported as being Duffy-negative homozygous in Harar and 24/107 (22%) in Jimma. Three *P. vivax-*positive individuals from Harar were Duffy-negative.	2013	[77]
738 patients from Anajás, Marajó Archipelago, State of Pará, Eastern Brazilian Amazon	Microscopy, real-time PCR, sequencing	PCR	Malaria was detected in 137 samples (20.2%) from the Eastern Brazilian Amazon. *P. vivax* prevalence was 13.9% (94/678), *P. falciparum* 5.8% (39/678) and *P. vivax* + *P. falciparum* 0.6% (4/678). 4.3% (29/678) were genotyped as Duffy-negative. 6.9% (2/29) Duffy-negative individuals were *P. vivax-*positive.	2012	[78]
995 individuals from Angola (n = 898) and Equatorial Guinea (n = 97)	Nested PCR	PCR-RFLP, sequencing	*P. vivax* was detected in 15 individuals, from which five exhibited a single *P. vixax* infection. All 15 samples were genotyped as Duffy-negative.	2011	[79]
11 participants from Kenya	Microscopy, PCR	Flow cytometry for Fy6 and Fy3 epitopes	*P. vivax* malaria was detected in 9 out of 11 Duffy-negative Kenyan individuals.	2006	[51]

## 6. *Plasmodium vivax* and Proteomics

Analysing the *P. vivax* Mrz reticulocyte infection stages in natural isolates represents a significant methodological challenge due to low parasitaemia, asynchronous parasite populations and frequent polyclonal infections. Proteome studies are expected to reveal further details regarding the parasite’s invasion and improve the understanding of its biology.

The first clinical proteome study, using isolated blood samples infected with early *P. vivax* stages, revealed 16 proteins [80]. The same authors were the first to examine the *P. vivax* proteome directly from a pool of clinical isolates in 2011; they identified 153 *P. vivax* proteins, of which ~36% were hypothetical proteins, 22 had metabolic functions, 14 chaperoning functions and 13 were involved in translation [81]. Studying the proteome by liquid chromatography with tandem mass spectrometry (LC-MS/MS) of schizont-enriched samples in the same year led to the identification of 316 proteins: ~50% were hypothetical proteins, 10% had binding functions, 5% were involved in the fate of proteins, 4% in protein synthesis, 4% in metabolism and 4% in cellular transport [82].

A study by our group using blood samples from a non-human primate model infected with *P. vivax* strain VCG-1 identified 329 proteins from ring stage-enriched, 238 from trophozoite-enriched and 727 from schizont-enriched samples, respectively [83]. LC-MS/MS has been used more recently for studying samples of infected *Saimiri boliviensis* reticulocytes in late trophozoite and early schizont stages; 2000 *P. vivax* proteins were identified, 25% of which lacked functional annotation [84].

Proteomics has contributed significantly to understanding the parasite’s biology. However, it also highlighted a large number of proteins expressed by the parasite whose function and potential interaction with receptors on host cells are unknown. Here, once again, everything falls back on the lack of a continuous in vitro culture system, which is a severe obstacle to the advancement of scientific knowledge of the parasite’s pathobiology. Nevertheless, rapid advances in LC MS/MS technology and the development of highly sensitive instruments/tools, such as single-cell proteomic (SCP) techniques, represent novel approaches to elucidate unknown molecular mechanisms of the parasite [85].

## 7. Conclusions

*P. vivax* is the most widely distributed parasite worldwide that causes malaria in humans. However, *P. vivax* malaria has been classified as a neglected tropical disease in terms of the development of specific therapies for its control or eradication. Significant advances have been impeded by a lack of or limited access to animal study models and the preferential infection of reticulocytes, which represent only 1–2% of total RBCs in the peripheral blood, thereby making the development of a continuous in vitro culture system an enormous challenge.

Three interactions that are likely involved in the parasite’s invasion of host cells have been characterised so far (*Pv*RBP2b-TfR1, *Pv*DBP-DARC and *Pv*RBP2a-CD98). TfR1 and CD98 are membrane proteins that are gradually lost during erythrocyte formation. It has been hypothesised that a mechanism by which *P. vivax* accurately targets which reticulocytes to invade involves TfR1 and CD98; however, the question whether additional reticulocyte-binding ligands also participate requires further study; in support of this hypothesis, other *P. vivax* proteins displaying preferential binding to reticulocytes have been described (e.g., *Pv*RBSA [86]). In contrast to TfR1 and CD98, the DARC is present in both reticulocytes and mature RBCs. It was believed that the DARC-negative population did not suffer the disease; however, many cases of *P. vivax* infection in this population have been reported during the last decade. Conventional rapid diagnostic tests (RDTs) and microscopy are the primary diagnostic tools to confirm suspected malaria. However, DARC-negative individuals are characterised by low parasitaemia and can be easily missed by the conventional techniques.

Although the Duffy-negative phenotype cannot be considered as a fully protecting factor, it explains why there are fewer cases of *P. vivax* in African countries, where it is predominant. Hypotheses have been proposed explaining *P. vivax* infection in Duffy-negative populations, including leaky DARC expression due to remaining expression on Duffy-negative RBCs or the potential existence of alternative invasion pathways, as used by *P. falciparum*, a species for which more cellular and molecular invasion characteristics are already known. It is noteworthy that an in vitro culture system for *P. falciparum* was established more than 40 years ago. Therefore, alternative methodologies for implementing long-term continuous in vitro culture systems should be established to better understand the biology of *P. vivax*.

Furthermore, understanding how *P. vivax* recognises and enters host cells will provide opportunities for blocking invasion and stopping the blood-stage infection cycle. Hence, functional studies are needed to identify the role of each parasite ligand involved in the invasion of Duffy-positive and Duffy-negative reticulocytes. Proteomics represents a useful tool for revealing unknown proteins expressed by the parasite during RBC invasion and revealing new pathways involved in parasite pathophysiology, which will lead to identifying new targets for developing methods for controlling or eradicating the disease.
